# Detection of human papillomavirus DNA in peri-tumor tissues and pelvic lymph nodes as potential molecular marker of micrometastasis in cervical cancer

**DOI:** 10.1186/s13027-016-0068-7

**Published:** 2016-05-11

**Authors:** Marianna Tortora, Clorinda Annunziata, Giuseppina Liguori, Simona Losito, Gerardo Botti, Stefano Greggi, Luigi Buonaguro, Franco M. Buonaguro, Maria Lina Tornesello

**Affiliations:** Molecular Biology and Viral Oncology Division, Istituto Nazionale Tumori “Fond. Pascale” - IRCCS, 80131 Napoli, Italy; Department of Pathology, Istituto Nazionale Tumori “Fond. Pascale” - IRCCS, Napoli, 80131 Italy; Division of Gynecology, Istituto Nazionale Tumori “Fond. Pascale” - IRCCS, Napoli, 80131 Italy

**Keywords:** HPV, Lymph nodes, Cervical cancer

## Abstract

**Background:**

The association between high risk human papillomaviruses (HPV) and cervical cancer has been firmly established. HPV genome is present in nearly all cases of cervical cancer and detection of viral DNA could therefore be used as a surrogate marker of micrometastasis in peri-tumor tissues and lymph nodes.

**Methods:**

We analyzed primary cervical carcinomas, peri-tumor biopsies and pelvic lymph nodes in 20 women with invasive cancer (FIGO stage I-II) who underwent radical pelvic surgery and lymphadenectomy. HPV DNA was searched by broad spectrum PCR in 142 DNA samples extracted from paraffin embedded tissues. Viral genotypes were identified by direct sequencing analysis.

**Results:**

HPV DNA sequences were identified in all available primary cervical tumors (*n* = 15). The most common genotype was HPV16 (60 %), followed by HPV18 (20 %), HPV35 (7 %), HPV45 (7 %) and HPV66 (7 %). Seven out of 20 (35 %) women had metastatic spread in peri-tumor tissues and/or lymph nodes, as determined by histology. HPV DNA was detected in all histological positive samples as well as in 16 and 25 % of histological negative peri-tumor tissues and lymph nodes, respectively. Three out of 20 (15 %) women without histological evidence of metastatic spread had HPV-positive lymph nodes. HPV genotype was found always concordant between primary tumor and metastatic lesions. The remaining 10 women (50 %) were histology and HPV-negative in all peri-tumor biopsies and lymph nodes analyzed.

**Conclusions:**

Evaluation of HPV DNA in peri-tumor tissues as well as pelvic lymph nodes could be a sensitive marker to identify micrometastasis or isolated tumor cells and to monitor the risk of disease recurrence in women with cervical cancer.

## Background

Cervical cancer is one of the most common tumors in women with 528,000 new cases and 266,000 deaths worldwide in 2012 [1]. The most frequent histological types are squamous cell carcinoma and adenocarcinoma, representing 85 % and 10–15 % of all cervical cancers, respectively.

Nearly all cases of cervical squamous cell carcinoma and adenocarcinoma are attributable to persistent infection with one of 12 high risk human papillomavirus (HPV) genotypes, namely HPV16, 18, 31, 33, 35, 39, 45, 51, 52, 56, 58 and 59 [2, 3]. HPV-related carcinogenesis is a long-term multistep process characterized by constitutive expression of early viral genes E6 and E7, which inactivate p53 and pRb oncosuppressors, respectively, causing deregulation of several cellular pathways and genomic instability [4].

Specifically, the E6 protein of high risk HPVs has shown to target p53 for fast proteasome-mediated degradation causing abrogation of cell growth-arrest and apoptosis [5, 6]. The E7 protein is able to bind and inactivate pRb with a consequent increase in free E2F, leading to both an increase in cyclin-dependent kinase inhibitor p16 and abnormal cell proliferation [7–9].

A crucial event in cervical carcinogenesis is represented by the integration of HPV genome into human chromosomes [10, 11]. DNA replication of HPV integrants has been shown to cause multiple genetic alterations such as formation of viral-host DNA concatemers, disruption of chromosomal regions and amplification of HPV E6 and E7 genes [12]. Viral sequences have been found frequently inserted in some hot spots such as 3q28, 8q24.21 and 13q22 loci [13], involving transcriptionally active regions [14], and preferentially within (34 %) or adjacent to fragile sites (32 %) [14–16]. The most frequently affected genes in either cervical intraepithelial neoplasia (CIN) or cancers are POU5F1B (9.7 %), FHIT (8.7 %), KLF12 (7.8 %), KLF5 (6.8 %), LRP1B (5.8 %), HMGA2 (7.8 %), and SEMA3D (4.9 %), supporting their oncogenic role in the clonal selection and outgrowth of neoplastic cells in the early stages of cervical carcinogenesis [17].

Standard treatment of cervical cancer is radical hysterectomy, bilateral adnexectomy and systematic pelvic lymph nodes removal [18, 19]. The histological type, tumor size, depth of invasion, lymphovascular space invasion, paracervical involvement and lymph nodal status represent the most important parameters for prognosis and choice of therapy [18, 20].

The metastatic spread of cervical carcinoma to pelvic lymph-nodes has been found in 0–29.3 % of women with early stages cervical cancer (FIGO IA1–IB1) and in 12–61.8 % of women with locally advanced disease (FIGO IB2–IIB) [21, 22]. Nonetheless, a recurrence rate of 10–15 % has been reported in patients with non-metastatic lymph nodes, probably due to histologically undetectable micrometastases or single tumor cells in the lymphatic system [21, 22]. Strander et al. showed that the long term risk of cancer among Swedish women treated for cervical intraepithelial neoplasia grade 3 (CIN3) was 2.5 fold higher for the development of cervical and vaginal carcinoma, compared with the general female population [23]. The risk of metachronous genital cancer lesions was higher in women treated over the age of 50 and persisted for more than 25 years after the initial treatment [23].

Vinokurova et al. [24] analyzed the HPV integration sites in multiple vaginal or vulvar metachronous lesions arising in women previously treated (up to 10 years before) for high grade cervical lesions or carcinomas, with resection margins reported to be disease free, and identified the same HPV integration sites in the primary as well in metachronous lesions from the same patient. These data support the hypothesis that vulvar and vaginal lesions were likely derived from locally disseminated dormant neoplastic cells, not detectable by histological analysis, raising the need of detecting HPV DNA by ultrasensitive molecular techniques in pelvic lymph nodes and peri-tumor tissues [25].

The aim of this study was to analyze the HPV DNA sequences in cervical carcinoma biopsies, peri-tumor tissues and pelvic lymph nodes to assess the sensitivity of molecular PCR method in identifying early metastasis or isolated cancer cells compared to histology.

## Methods

### Patients and tissue samples

One hundred forty-two formalin-fixed and paraffin-embedded biopsies from 20 patients referred to the Gynecology Unit at the National Cancer Institute Fond Pascale were included in the study. All enrolled patients underwent surgical treatment for the removal of primary tumor, adjacent tissues biopsies and pelvic lymph nodes and histology of all samples was determined. All patients with locally advanced cervical cancer (stages IB2 or worse) received standard neo-adjuvant chemotherapy before surgery. To perform molecular analysis, four 10-μm thick sections from each paraffin block were obtained in separate sterile Eppendorf tubes for PCR analysis and two sections for hematoxylin–eosin staining. Slides immediately adjacent to the tissue sections used for viral DNA analysis were reviewed by the pathologist to verify the presence of neoplastic tissue. Histological subtypes were determined in accordance to Young et al. [26] and to the Bethesda 2001 system [27]. Tissues were graded as adenocarcinoma (endometrioid, clear cell and serous types, *n* = 4), and invasive squamous cell carcinoma (*n* = 16). This study was approved by the Institutional Scientific Board and by the Ethical Committee of the Istituto Nazionale Tumori “Fond Pascale”, and is in accordance with the principles of the Declaration of Helsinki.

### DNA isolation

Genomic DNA was extracted according to published procedures [28]. In particular tissue samples were digested over night at 56 °C with Proteinase K (200 μg per ml) in 100 μl of lysis buffer (50 mM Tris-HCl pH 8.5, 1 mM EDTA, 0.5 % Tween20), followed by DNA purification by phenol and phenol-chloroform-isoamyl alcohol (25:24:1) extraction and ethanol precipitation in 0.3 M sodium acetate (pH 4.6).

### PCR amplification

The concentration of all manually extracted DNA samples was evaluated by spectrophotometry (NanoDrop™ 2000). The quality of nucleic acid was determined by PCR amplification of a 150 bp fragment within the exon 7 of *TP53* gene [29]. HPV detection was carried out by nested PCR with MY09/MY11 primer pairs [30] for the outer reaction and MGP primer system for the inner reaction in 50 μl reaction mixture containing 5 μl of outer reaction, as described previously [31]. This PCR system was evaluated for its sensitivity and specificity for individual HPV types by using a proficiency panel of HPV plasmids in serial dilutions, both alone and in combination, obtained in the context of the 4th WHO HPV LabNet Proficiency Study for Evaluating HPV DNA Typing Methods (2010). The system was evaluated as proficient for detection of HPV 16, 18, 31, 33, 35, 39, 45, 52, 56, 58, 59, 66 and 68b, being able to detect 50 genome equivalents (GE)/5 μl of HPV 16 and HPV 18 DNA, and 500 GE/5 μl of the other HPV types with a specificity above 97 % [32]. A reaction mixture without template DNA, as negative control, was included in every set of five clinical specimens for each PCR run.

The amplification products were subjected to electrophoresis on a 7 % polyacrylamide gel followed by staining with ethidium bromide. The image was scanned with the system image capture Gel Doc (Bio-Rad). HPV genotypes were identified by direct automated DNA sequencing analysis of MGP amplified products using both the forward GP5+ and reverse GP6+ oligoprimers [33] at Eurofins Laboratories (Milan). HPV type identification was performed by alignments of HPV sequences with those present in the GenBank database using the BLASTn software (http://www.ncbi.nlm.nih.gov/blast/html).

#### Statistical analyses

The data were analyzed with Epi Info 6 Statistical Analysis System Software (Version 6.04b, 1997, Centers for Disease Control and Prevention, USA). Unpaired *t* test was used for comparisons of continuous variables (i.e. age); Yates-corrected *χ*^2^ test and, where appropriate, two-sided Fisher’s exact test were used for comparison of categorical data. Differences were considered to be statistically significant when *P* values were less than 0.05.

## Results

The study included 20 women with a mean age at diagnosis of 48.3 (±11.84) years. According to FIGO staging the study included four IA (20 %), ten IB (50 %), three IIA (15 %), two IIB (10 %) and one IIIB (5 %) stage patients (Table 1). Sixteen (80 %) cases were diagnosed as squamous cell carcinoma (SCC) and four (20 %) as adenocarcinoma (AC) of the cervix.

**Table 1:** Clinic-pathological characteristics of patients with cervical cancer

	Total cases (*n* = 20)	Metastatic cases (*n* = 7)
Mean age (±SD)	48. 3 (±11,84)	45.86 (±12.9)
≤ 45	10	4 (57.2 %)
> 45	10	3 (42.8 %)
FIGO Stage		
I A	4 (20 %)	0 -
I B	10 (50 %)	4 (57.1 %)
II A	3 (15 %)	1 (14.3 %)
II B	2 (10 %)	1 (14.3 %)
III B	1 (5 %)	1 (14.3 %)
Histopathology		
Squamous cell carcinoma (SCC)	16 (80 %)	6 (85.8 %)
Adenocarcinoma (AC)	4 (20 %)	1 (14.2 %)
Grading		
Well differenziated (G1)	2 (10 %)	1 (14.2 %)
Moderately differentiated (G2)	6 (30 %)	1 (14.2 %)
Poorly differentiated (G3)	12 (60 %)	5 (71.6 %)
Volume of primary lesion		
< 2 cm	4 (20 %)	0 -
2-4 cm	3 (15 %)	2 (28.6 %)
> 4	2 (10 %)	2 (28.6 %)
Tumor Invasion		
Peri-tumor tissues	3 (15 %)	3 (42.9 %)
Lymph nodes	3 (15 %)	3 (42.9 %)
Peri-tumor + Lymph nodes	1 (5 %)	1 (14.2 %)

All tissue samples obtained from each woman were grouped in 1) primary tumors, 2) peri-tumor tissues and 3) pelvic lymph node samples, each consisting of a lymph node station comprising between 2 and 25 lymph nodes in one or more inclusions. In total 142 tissue samples were analyzed of which 15 primary tumors, 54 peri-tumor tissues and 73 lymph node inclusions for a total of 304 lymph nodes. Samples from primary tumor biopsies were not available in five patients.

Seven out of 20 (35 %) women had metastatic spread in peri-tumor tissues and/or lymph nodes, as determined by histology. In particular, three patients (43 %) had pelvic lymph nodes metastases, three (43 %) peri-tumor invasion and one patient (14 %) had both peri-tumor and pelvic lymph nodes metastases.

The distribution of viral genotypes is shown in Table 2. Overall, HPV DNA sequences were detected in 63 out of 142 (44.4 %) samples. The most common HPV genotype was HPV16 (51/63, 81 %), followed by HPV18 (4/63, 6.3 %), HPV45 (4/63, 6.3 %), HPV35 (2/63, 3.2 %) and HPV66 (2/63, 3.2 %). All available primary tumor samples were positive for HPV DNA sequences. The most common genotype was HPV16 (9/15, 60 %), followed by HPV18 (3/15, 20 %), HPV35 (1/15, 7 %), HPV45 (1/15, 7 %) and HPV66 (1/15, 7 %). All histological positive peri-tumor tissues (*n* = 23) and lymph nodes (*n* = 3 groups) were positive for HPV DNA sequences. Moreover, viral DNA was detected in 5 out of 31 peri-tumor tissues and in 17 out of 68 lymph nodes groups (comprising a total of 182 lymph nodes) which were histological negative for micrometastasis (Tables 2 and 3). There was no statistically significant difference in the frequency of HPV positive lymph nodes (*p* = 0.94) or peri-tumor tissues (*p* = 0.63) according to FIGO stages. However, bias could have occurred due to the small number of patients in some FIGO groups.

**Table 2:** Presence of HPV DNA in histological positive and negative peri-tumor tissues and lymph nodes from cervical cancer patients

Cases	Histol	Grade	FIGO Stage	Metast. Peri-tumor (%)	Metastatic LN (%)^a^	HPV-pos Peri-tumor (%)	HPV-pos LN (%)	HPV^b^	Recurrence^c^
SCC-04	SCC	3	IB	3/6 (50)	0/4 -	4/6 (66.6)	2/4 (50)	HPV16	Pelvic
SCC-05	SCC	2	IA	0/2 -	0/1 -	0/2 -	0/1 -	HPV18	No
SCC-07	SCC	3	IIIB	1/2 (50)	0 -	1/2 (50)	0 -	HPV35	Lung
SCC-09	SCC	3	IA	0/4 -	0/1 -	0/4 -	0/1 -	n/a	No
SCC-12	SCC	2	IIA	0 -	0/1 -	0 -	0/1 -	HPV16	Pelvic/Lung
SCC-20	SCC	3	IA	0/3 -	0/2 -	0/3 -	0/2 -	n/a	No
SCC-22	SCC	2	IB	0/2 -	0/3 -	1/2 (50)	2/3 (66.6)	HPV45	No
SCC-24	SCC	1	IB	0/2 -	0/6 -	1/2 (50)	5/6 (83.3)	HPV16	n/a
SCC-39	SCC	3	IIB	0 -	1/4 (25)	0 -	3/4 (75)	HPV16	Lung
SCC-41	SCC	3	IB	0/3 -	2/3 (66.6)	1/3 (33.3)	0/3 -	HPV66	No
SCC-42	SCC	2	IA	0 -	0/3 -	0 -	0/3 -	n/a	No
SCC-43	SCC	3	IB	0 -	0/9 -	0 -	0/9 -	n/a	No
SCC-44	SCC	3	IB	8/9 (88.8)	0/6 -	8/9 (88.8)	6/6 (100)	HPV16	n/a
SCC-45	SCC	1	IB	11/11 (100)	1/10 (10)	11/11 (100)	1/10 (10)	HPV16	n/a
SCC-46	SCC	3	IB	0 -	0/8 -	0 -	0/8 -	HPV16	n/a
SCC-47	SCC	3	IIB	0 -	0/2 -	0 -	0/2 -	HPV16	Dead
AC-10	AC	3	IB	0/5 -	0/3 -	1/5 (20)	0/3 -	HPV16	n/a
AC-11	AC	2	IIA	0/1 -	1/4 (25)	0/1 -	1/4 (25)	HPV18	n/a
AC-18	AC	3	IB	0/4 -	0/2 -	0/4 -	0/2 -	n/a	No
AC-19	AC	2	IIA	0 -	0/1 -	0 -	0/1 -	HPV18	Pelvic

^a^ Each lymph node inclusion comprises 2–25 lymph nodes for a total of 304 lymph nodes

^b^ n/a - primary tumor was not available

^c^ n/a - Information on disease recurrence was not available; No- no disease recurrence, Local - loco regional recurrence, Distant - metastasis outside the pelvis

**Table 3:** Comparison between histology and PCR results

	HPV PCR pos (%)	HPV PCR neg (%)	*P* value^a^
Primary tumor			
Histology pos (%)	15 (75)	0	
Peri-tumor tissues			
Histology pos (%)	23 (42.6)	0	
Histology neg (%)	5 (9.3)	26 (48.1)	<0.0001
Lymph-nodes			
Histology pos (%)	3 (4.1)	2 (2.7)	
Histology neg (%)	17 (23.3)	51 (69.9)	0.2403

^a^ Yates-correction *χ* ^2^ test

During a 24 months follow-up period one patient died and 5 experienced disease recurrence: two patients (SCC-04 and AC-19, positive for HPV16 and 18, respectively) had loco-regional recurrence, two (SCC-07 and SCC-39, positive for HPV35 and 16, respectively) were diagnosed with distant metastasis outside the pelvis and one patient (SCC-12, positive HPV16) was diagnosed with both loco-regional and distant metastases (Table 2). Two patients (SCC-22 and SCC-41, positive for HPV45 and 66, respectively) with histological negative/HPV-positive peri-tumor and/or lymph nodes samples had no recurrence during the follow up. Information on disease recurrence was not available for the remaining 6 patients.

**Fig. 1:**
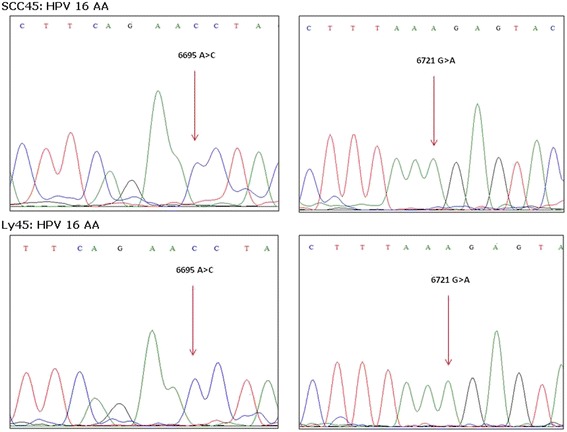
DNA sequence electropherograms showing HPV 16 Asian–American nucleotide signatures in L1 region at nucleotide position 6695 (A > C) and 6721 (G > A) in the primary tumor (SCC45) and metastatic lymph node (Ly45)

All HPV genotypes identified in peri-tumor samples and lymph nodes were concordant with that identified in the corresponding primary tumor. Moreover in one case the HPV16 DNA sequences amplified in the primary tumor (SCC-45) and corresponding lymph nodes (LN45), both contained two nucleotide changes (nt 6695 [A > C] and 6721 [G > A]) distinctive of Asian-American variants, supporting the clonality of tumor cells (Fig. 1).

## Discussion

Lymph node status in women with cervical cancer is the most important parameter for predicting outcome of disease and for planning post-surgery adjuvant therapy [18]. However, a significant proportion of women, treated for early stage cervical cancer and without nodal metastasis, develop local or distant disease up to decades after successful treatment of primary lesions [23]. Balamurugan et al. analyzed the Epidemiology and End Results (SEER) cancer data, collected during the period 1992–2004, and observed a higher risk of in situ and invasive cancers of the vagina (standardized incidence ratios, SIRs of 53.8 and SIRs of 29.9, respectively) and vulva (SIRs of 6.6 and 5.7 for invasive cancer, respectively) among cervical cancer survivors [34].

The analysis of HPV DNA, generally integrated within the genome of the host cancer cells, could represent a sensitive marker of micrometastases and single tumor cell spread in pathologically-negative peri-tumor tissues and lymph nodes [25]. Since the first report by Lancaster et al. [35] on the utility of HPV DNA detection in lymph nodes as surrogate marker of cervical cancer metastasis, several retrospective and prospective studies were conducted with the aim to validate the clinical usefulness of this analysis, often with divergent results [25].

In the current retrospective study, we have identified HPV sequences in all histological positive samples as well as in 16 and 25 % of histological negative peri-tumor tissues and lymph nodes, respectively. Among the seven women with histological evidence of metastatic spread the number of HPV positive lymph nodes and/or peri-tumor tissues was much higher compared to histological analysis. Three out of 20 (15 %) women had at least an HPV-positive lymph node sample without histological evidence of metastatic spread. Our results are in agreement with several published studies. In particular, Noventa et al. [25] in the systematic review including 15 studies on the histology and HPV detection in lymph nodes among 1333 patients with early stage cervical cancer, showed that HPV DNA was present in 75 % of cases with at least one lymph node metastasis (488 women) and in 39 % of cases without metastatic involvement (913 women). Higher rates of HPV positivity have been reported by Slama et al. [36] which, based on their work on fresh tissues, found viral genomes in 66.6 % of histological negative lymph nodes from patients without metastatic involvement.

Several studies showed that presence of HPV-DNA in lymph nodes is associated with increased risk of disease recurrence and reduced overall survival [37–39]. Others reported no significant differences in recurrence or overall survival between patients with positive and negative HPV-DNA lymph nodal status independently from the histology [40, 41]. Lukaszuk et al. [42] conducted the first prospective study on frozen fresh-tissues of cervical lesions and pelvic lymph nodes and demonstrated that viral DNA in lymph nodes was an independent oncological risk factor, correlating with survival and mortality rate. More recently, Durst et al. [43] evaluated the expression of HPV E6/E7 mRNA as a molecular marker for the detection of tumor cells in fresh biopsies of histological negative sentinel lymph nodes and showed that recurrence-free survival was significantly longer for patients with HPV mRNA negative sentinel lymph nodes (log rank *p* = 0.002).

In our study the most representative viral genotypes in all HPV positive tissues were HPV16 (81 %), followed by HPV18 (6.3 %), HPV45 (6.3 %), HPV35 (3.2 %) and HPV66 (3.2 %). The HPV genotype was always concordant between primary tumor and metastatic/non metastatic HPV-positive lesions from the same patient. Accordingly, Landro et al. [41] reported a high prevalence of HPV16 and HPV18 in primary lesions (84 and 27 % positive, respectively) and lymph nodes (46 and 20 %, respectively). In their study the correspondence of viral genome between primary lesion and lymph nodes was 73 %, while in 13.5 % of cases the viral sequences were not concordant and in 13.5 % the HPV DNA was not detected [41]. In fact, it is expected that in some cases the HPV positivity is due to the ability of immune-competent phagocytes to transport the HPV-positive cells and/or viral particles from the primary tumor to lymph nodes. Landro et al. [41], observed that in metastases-free lymph nodes the HPV sequences were evident by PCR and in situ hybridization in nuclei and/or cytoplasm of lymphocytes from germinal centers or cortical areas, in endothelial cells, in macrophages and in stromal cells. In metastatic lymph nodes the HPV DNA was detected in all the previous mentioned cells and also in squamous invasive cells.

The main limitations of our study include its retrospective design, the inability to evaluate long term disease recurrence of these women, and the small number of patients, although the number of samples analyzed for each patient was relatively high.

## Conclusions

In conclusion, our data suggest that the analysis of HPV sequences is more sensitive compared to histological analysis. The clinical implications of HPV DNA sequences in peri-tumor tissues and lymph nodes and the benefit for the patients with respect to prediction of disease recurrence and therapy options will have to be addressed in further prospective studies.

## Consent

 All patients provided written informed consent.

## Abbreviations

AC: adenocarcinoma
HPV: human papillomavirus
PCR: polymerase chain reaction
SCC: squamous cell carcinoma
